# Experimental study of the mutual interactions between waves and tailored turbulence

**DOI:** 10.1017/jfm.2023.280

**Published:** 2023-05-10

**Authors:** Benjamin K. Smeltzer, Olav Rømcke, R. Jason Hearst, Simen Å. Ellingsen

**Affiliations:** 1 Department of Energy and Process Engineering, Norwegian University of Science and Technology, N-7491, Trondheim, Norway; 2 SINTEF Ocean, Marinteknisk senter, N-7052 Trondheim, Norway

**Keywords:** Keywords to be entered during submission process

## Abstract

When surface waves interact with ambient turbulence, the two affect each other mutually. Turbulent eddies get redirected, intensified and periodically stretched and compressed, while the waves suffer directional scattering. We study these mutual interactions experimentally in the water channel laboratory at the Norwegian University of Science and Technology (NTNU) Trondheim. Long groups of waves were propagated upstream on currents with identical mean flow but different turbulence properties, created by an active grid at the current inlet. The subsurface flow in the spanwise–vertical plane was measured with stereo particle-image velocimetry. Comparing the subsurface velocity fields before and after the passage of a wave group, a strong enhancement of streamwise vorticity is observed which increases rapidly towards the surface for *k*_0_z ≳ -0.3 (*z*: vertical distance from still surface; *k*_0_: carrier wavenumber) in qualitative agreement with theory. Next, we measure the broadening of the directional wave spectrum at increasing propagation distance. The rate of directional diffusion is greatest for the turbulent case with the highest energy at the longest length scales whereas the highest total turbulent kinetic energy overall did not produce the most scattering. The variance of directional spectra is found to increase linearly as a function of propagation time.

## 1 Introduction

Surface waves in environmental flows almost invariably coexist with ambient turbulence in the water phase. The turbulent motion just beneath the surface controls the flux of gas across the ocean-atmosphere interface (e.g. Wanninkhof *et al*. 2009), the fate of pollutants such as microplastics (e.g., Jalón-Rojas *et al*. 2019) and the motion, composition and blooming of phytoplankton (Durham *et al*. 2013). There are several mechanisms by which turbulence and waves affect each other mutually, predicted and confirmed as reviewed below, yet little empirical evidence exists quantifying how these mutual interactions depend on the properties of the ambient turbulence. In the present study we investigate this question experimentally in a laboratory where the turbulence beneath the waves can be tailored directly.

The most immediate effect of waves on turbulence — of linear order in wave steepness *ϵ* = *k*_0_*a* according to theory where *k*_0_ and *a* are wavenumber and amplitude, respectively — is periodical stretching and compression of streamwise turbulent eddies beneath troughs and crests, respectively. The phenomenon has been strikingly visualised (e.g. Veron *et al*. 2009; Savelyev *et al*. 2020) and through simulations, Guo & Shen (2013, 2014) and Xuan *et al*. (2019) provide a deterministic picture with corresponding intensification and relaxation of streamwise vorticity. Previous measurements of turbulence variation with wave phase have not measured vorticity to our knowledge, but rather different Reynolds stress components beneath waves, most finding diagonal components to be enhanced (reduced) beneath troughs (crests) (Jiang & Street 1991; Thais & Magnaudet 1996). Curiously, Savelyev *et al*. (2012) appear to find the opposite trend – a peak in horizontal contributions to turbulent kinetic energy (TKE) beneath crests which we cannot explain. (As do Rashidi *et al*. (1992), but attribute it to boundary-layer interactions, absent in our set-up). Note, however, that the vortex stretching process intensifies streamwise vorticity, whose relation to TKE is non-local through the Biot-Savart law: stronger vorticity tends to imply an increase in kinetic energy, but not in general at the same spatial location. Herein, we measure the streamwise vorticity itself, a more direct and arguably more clear-cut test. Unlike Reynolds stress, taking vorticity as our measured quantity eschews the highly non-trivial task of separating turbulent from wave-orbital motion, since time variations in vorticity can be uniquely ascribed to turbulence, not waves.

A less conspicuous interaction is also present, scaling in theory (Teixeira & Belcher 2002) as *ϵ*^2^*t* where *t* is time, driven by a coupling between the wave’s induced Stokes drift and turbulent eddies, whereby the vertical vorticity of eddies is tilted into the direction of wave propagation and intensified through stretching. Although of second order, the effect accumulates in time and can become highly significant. Early experiments showed that mechanically generated waves increased turbulence intensity (Van Hoften & Karaki 1977; Cheung & Street 1988), and Teixeira & Belcher (2002) predicted how a passing wave transfers energy to ambient turbulence and modifies Reynolds stresses anisotropically. McWilliams *et al*. (1997) and Ardhuin & Jenkins (2006) found by different theoretical approaches that waves, on average, produce TKE as though the Stokes drift velocity were an Eulerian shear current. Increased TKE must be accompanied by a corresponding decrease of wave energy; Teixeira (2012) argued that this mechanism could explain surprisingly high levels of wave dissipation observed in field studies (e.g. Ardhuin *et al*. 2009). Experimental observations of turbulence intensification under non-breaking waves has been reported by Thais & Magnaudet (1996) and Savelyev *et al*. (2012), who both observed the formation and intensification of streamwise vortices, stronger for higher wave steepnesses, in qualitative agreement with theory predictions.

We emphasise that what we consider is waves interacting with ambient grid turbulence, distinct from the phenomenon of Langmuir turbulence often associated with wave-current interactions. The two differ in their mechanism of generation: ‘regular’ turbulence is created in real flows from viscous stress at the flow boundaries (e.g. wind stress, wave breaking, precipitation or solid boundaries, in our case the active grid; in simulation a synthetic non-conservative bulk force is often used). Langmuir turbulence, on the other hand, develops from coherent Langmuir circulation rolls which develop through laminar mechanisms famously described by Craik and Leibovich (Leibovich 1983). A connection between the two might be argued (see discussions, e.g. in Teixeira & Belcher 2010; Teixeira 2012; Guo & Shen 2013) because after the passage of waves, the initially near-isotropic turbulence has developed long streamwise vortical structures which, say Peruzzi *et al*. (2021), “share many features with Langmuir-type cells”. Traditionally the two are considered distinct because these vortices extract their streamwise vorticity from the disordered ambient turbulence whereas Langmuir circulation takes theirs from the mean flow. A further discussion of this fascinating point is beyond the scope of our investigation.

Conversely, waves are affected by the presence of background turbulence. A number of studies have focused on wave refraction from “macroturbulence” (e.g., White & Fornberg 1998; Villas Bôas & Young 2020; Smit & Janssen 2019), random currents varying slowly on the scale of a wavelength, in which case a geometrical optics approximation can be employed.

When the length scales of the turbulent motion are allowed to be the same order of magnitude as the wavelength, however, theory is scarce. Phillips (1959), and later Fabrikant & Raevsky (1994), derived expressions for the scattering of linear waves impinging on a random distribution of vorticity; although illuminating, these are hard to apply in practice since they require the spatial spectrum of the vorticity up to the largest turbulent scales, a very demanding measurement to make. In rough terms they both find that the rate of directional broadening due to linear, small-angle scattering events is proportional to the spatial correlations among vorticity components over distances of a wavelength or more (subject to assumptions, the scattering rate of a wave of wavenumber *k* at small angle *θ* is proportional to the spatial vorticity covariance spectrum at “vorticity wavenumber” *2k* sin(*θ*/2) (Phillips 1959)). On the other hand, the scattering rate is proportional to the integrated vorticity spatial (co)variance spectrum, hence might be expected to increase with increasing TKE. Notably, Bal & Chou (2002) derived scattering rates of waves on a rapidly varying random potential flow (they are careful not to refer to this as “turbulence”), obtaining results of a similar structure, now involving the correlation function among velocities rather than vorticities. Although difficult to compare, an indication is that scattering is primarily a process of diffraction and refraction due to velocity variations, not interaction with the vorticity directly.

In the following, we report on an experimental investigation of these mutual wave/turbulence interactions. With measurements of the streamwise vorticity as a function of depth we quantify the periodic turbulence stretching and compression under passing regular waves, and the cumulative intensification of turbulence caused by the passage of a wave group, in the presence of initial turbulence with disstinct physical properties. Conversely we measure how the angular distribution of propagating waves spread as a function of propagation distance for different turbulent flows and find that the scattering of waves by turbulence depends more on the presence of large turbulence structures than on the total TKE.

## 2 Experimental Methods

**Figure 1:**
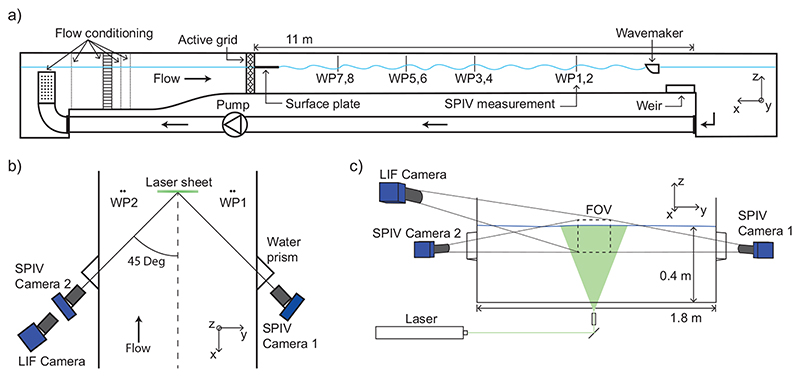
Experimental setup: (*a*) side view of water channel (WP: wave probe) with flow from left to right, (*b*) top view of measurement region, (*c*) longitudinal view.

The experiments were performed in the water channel facility at NTNU Trondheim. Water is recirculated through the test section of dimensions 11 m × 1.8 m × 1.0 m (length × width × height). Tailored turbulence was created by an active grid of diamond-shaped wings, mounted on 18 × 10 bars (vertical × horizontal, mesh length *M* = 10 cm), each controlled by a stepper motor. A diagram of the set-up is shown in figure 1; see Jooss *et al*. (2021) for further details.

A plunger wavemaker, 10.2 m = 102*M* from the grid, generated wave groups of centre frequency *f*_0_ = 1.02 Hz propagating upstream. A weir at the outlet accelerated the mean flow beyond the group velocity of relevant frequencies to remove reflections from the downstream wall. Waves of group velocity slower than the mean flow were unable to propagate upstream, including high-frequency wave noise from the wavemaker as well as free harmonics. The waves remained largely two-dimensional in all cases, i.e., no clearly visible 3-D instabilities manifested (see discussion by, e.g., Melville (1982)).

Boundary layers near the bottom and sidewalls are thin (momentum thickness ≲ 26 mm at the end of the channel, as reported by Jooss *et al*. (2021)), and the water is deep as seen by the waves, so essentially all turbulence which interacts with the waves in our experiment is created only by the grid, not boundary layers. We observe no sign of the steady, tank-wide Langmuir-type rolling motion observed in similar wave-current set-ups in narrower tanks; see the discussion by Groeneweg & Battjes (2003) and others referenced therein.

We denote the measured velocity field components in the {*x*, *y*, *z*} directions (with axes as defined in figure 1a) as {*u*, *v*, *w*} = {−*U*_0_ + *u*’, *v*’, *w*’} where *U*_0_ is the mean streamwise velocity and primed quantities are zero-mean variations from turbulence (and, when present, waves, but we never explicitly refer to wave orbital velocities herein). In the following, 〈 … 〉_*κ*_ denotes averaging with respect to a dimension *κ* which can be *y*, *z* or *t* or combinations of these. Ensemble averaging is understood for all averaged quantities. The root-mean-square (RMS) is denoted with subscript ‘∞’ and implies *u*_∞_ = (〈*u*’^2^〉_*yzt*_)^1/2^ etc., except where an explicit dependence on *z* is indicated in which case averaging is with respect to *y* and *t* only.

All three components of the velocity field were measured in a plane perpendicular to the mean flow 8.38 m = 83.8*M* downstream from the active grid, using stereoscopic particle image velocimetry (SPIV). Two 25 megapixel CMOS cameras (LaVision Imager MX 25M) were positioned on either side of the channel glass sidewalls at ±45° to the streamwise axis (see Fig. 1b), each fitted with a 180 mm focal length lens (Sigma). The output from a dual-head Nd:YAG laser (Litron Nano L 200-15 PIV) produced the SPIV light sheet illuminating 40 *μ*m polystyrene sphere seeding particles. LaVision DaVis version 10.1 was used to record and process the images. A multi-pass cross-correlation algorithm was used for processing the SPIV frames, with an initial pass of window size 128 × 128 pixels, and a final pass of 48 × 48 pixels (1.6 × 1.6 mm), both with 50 % overlap. The spacing of the output velocity vectors d*x* was thus 0.8 mm. The uncertainty in the measured velocities in the horizontal directions was roughly 3 mm/s using the method of Foucaut & Stanislas (2002) based on the velocity spectra. The uncertainty for the vertical velocity was roughly a factor two smaller.

We frequently report mean–square quantities. Assuming systematic biases are negligible, the measured and reported value of the mean square of some turbulent quantity *b*’ equals $b_{\infty }^{2}=\langle b{'}^{2}\rangle =\langle b_{\text{true}}^{'2}\rangle +\langle \varepsilon^{2}\rangle$ where $b_{\text{true}}^{'}$ is the true value and *ε* the uncertainty. The absolute value of mean-square values can thus have considerable uncertainty while differences in $b_{\infty }^{2}$ are relatively unaffected.

To detect and mask the moving free surface in the SPIV images, a laser induced fluorescence (LIF)-based technique was used following Buckley & Veron (2017). A small amount of rhodamine-6G was added to the water, causing water and air phases to appear light and dark to the LIF camera, respectively, with the surface readily identifiable as a sharp brightness transition. The combined particle-image velocimetry (PIV) and LIF system had a usable field of view of approximately 120 mm × 140 mm (width × height). A 4 × 2 grid of resistive wave probes (HR Wallingford) were mounted pairwise a spanwise distance Δ*y* = 120 cm apart (i.e., 30 cm from each channel wall), at streamwise locations 1.95 m, 3.80 m, 5.40m and 8.38m downstream from the active grid. These are labelled as WP1 to WP8 as shown in figure 1a and b.

The experiments consisted of generating wave groups propagating upstream atop the turbulent flows tailored by the active grid, and performing SPIV/LIF measurements with acquisition frequency 8Hz during three intervals with respect to each group. The experimental conditions are listed in table 1 where $\bar{f_{G}}$ is the mean active-grid frequency, *S*_0_ the wavemaker peak stroke, *τ*_wm_ the wave group temporal width as applied at the wavemaker, *T*_wm_ the duration of wavemaker actuation, *T*_PIV_ the duration of SPIV measurement for each interval, *N*_int_ the number of measurement intervals per wave group. Other quantities are defined below.

Four different active-grid actuation cases were investigated, labelled from *A* to *D*. The grid wings were rotated with random rotational velocity, acceleration and period within set limits (Hearst & Lavoie 2015). The instantaneous rotational velocity varied by $\pm 0.5\bar{f_{G}}$ with a top-hat distribution. In case *B*, only the vertically oriented grid bars were actuated. For case *A*, the grid was stationary with the wings aligned in the position of least blockage.

**Figure 2:**
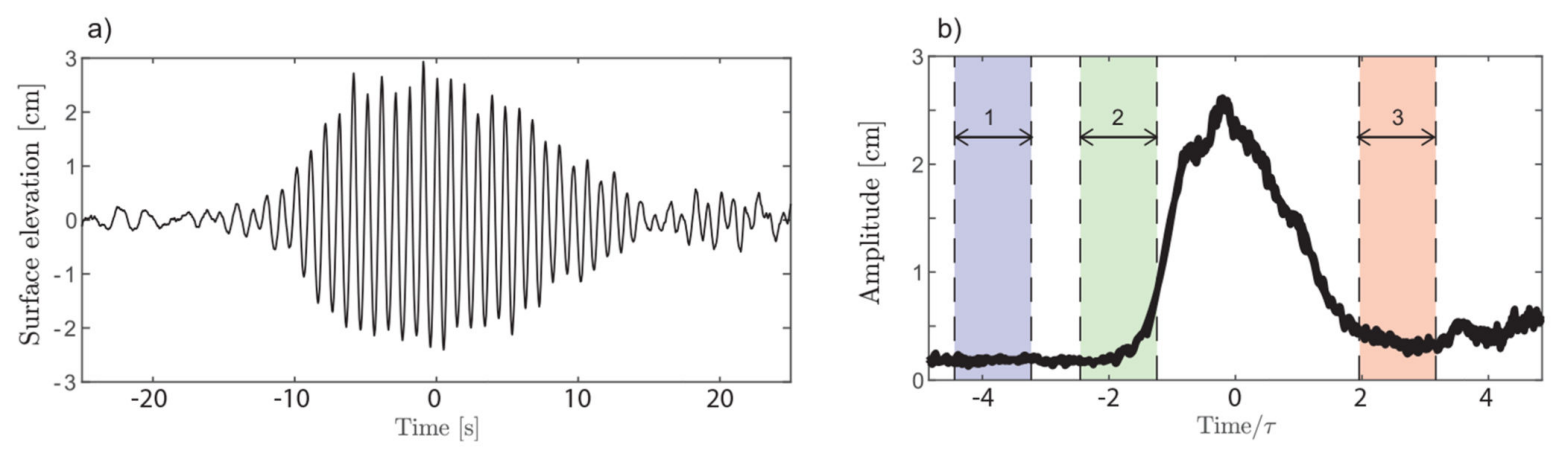
(*a*) Example surface elevation of a single wave group (case D), measured by a wave probe at the SPIV measurement location. (*b*) Ensemble-average group amplitude envelope as a function of time for case *A*. The time intervals for SPIV measurement are shaded.

The wave groups were generated with the wavemaker stroke *S*(*t*) having a Gaussian amplitude envelope of the form $S\left(t\right)=S_{0}\text{exp}\left[-\frac{1}{2}{\left(t-\frac{1}{2}T_{\text{wm}}\right)}^{2}/\tau_{\text{wm}}^{2}\right]$. The surface elevation for one wave group is shown for illustration in figure 2a). To alleviate modulational instability, the wavemaker’s actuation frequency was chirped to defocus the wave group.

For all test cases in table 1 with the exception of *C3*, SPIV/LIF measurements were performed during three intervals: well before the group arrived, and at the leading and trailing edges of the group envelope, respectively, as shown in figure 2b. Due to the counter-currents, the leading edge of the wave group takes nearly 40 s to reach the surface plate, which is after the end of interval 3. For *C3* where the group width was significantly longer and the wave amplitude approximately constant, a single measurement interval of longer duration was used. After each group, residual waves from reflections were allowed to dissipate for approximately five minutes before the next wave group was generated. The above procedure was performed a total *N*_ens_ times for ensemble statistics. Turbulent flow conditions without waves for cases *A*-*D* were further characterised from additional measurements consisting of four ensembles, each 67 s, sampled at 15 Hz.

## 3 Results

The measured physical characteristics of the mean flow, turbulence and waves are shown in table 2. The mean flow velocity magnitude *U*_0_ was approximately constant in the spanwise and vertical directions over the field of view. The water depth *h* ≈ 0.4 m was sufficient so that deep water could be assumed, hence *k*_0_ and *f*_0_ are related by $2\pi f_{0}=\sqrt{gk_{0}}-U_{0}k_{0}$ where *g* is the gravitational acceleration. The carrier wavelength is *λ*_0_ = 2*π*/*k*_0_.

The RMS of the turbulent velocity fluctuation prior to the wave groups was evaluated in interval 1. The quantity *u*_∞_/*U*_0_ is the streamwise turbulence intensity, etc. Note that in these coordinates, the waves propagate in the positive *x* direction. The length scale $L_{x}^{x}$ in table 2 is representative of the streamwise extent of the largest prevalent turbulent structures, defined in section 3.2.

Characteristic values for peak amplitude *a*_0_ and lab-frame group width *τ* were estimated as, respectively, the maximum and standard deviation when fitting a Gaussian to the average wave envelope measured by the probes WP1,2 (see figure 2b). The exception is case *C*3 where the average amplitude was used. The characteristic wave steepness is *ϵ* = *a*_0_*k*_0_. For a more direct comparison with other experiments and theory, an intrinsic group width *τ*_0_ is defined as *τ*_0_ = *τc_g_*/*c*_*g*0_, where $c_{g0}=\frac{1}{2}\sqrt{g/k_{0}}$ is the intrinsic group velocity and *c_g_* = *c*_*g*0_ − *U*_0_ is the group velocity in the lab system. The intrinsic group width reflects the time scale during which the ambient turbulence interacts with the wave group. The associated group length *α* = 4*τ*_0_*c*_*g*0_ defined as the full width of the group at 13.5% of the peak amplitude was ≈ 6m, about half the length of the test section.

### 3.1 Wave effects on the streamwise vorticity

To linear order in (presumably small)wave steepness, vortices are predicted to be periodically stretched and strained over the wave cycle with no change in strength on average. However, to second order in wave steepness, interactions with the Stokes drift results in a cumulative increase in streamwise vorticity over multiple wave cycles.

The streamwise vorticity component Ω’ = *∂_y_w*’ − *∂_z_v*’ was evaluated from the spatial gradients of the in-plane velocity components. The gradients were evaluated using a second-order finite difference scheme with a spacing of 6 velocity vectors, giving an uncertainty of *σ_∂x_* ∼ 0.45 s^-1^ estimated using the theory of Foucaut *et al*. (2021).

We refer to the quantity Ω^2^ = Ω^2^(*y*, *z*, *t*) as the cross–plane enstrophy. Angular brackets with subscript ‘*φ*’ denote the phase average, ${\langle \cdots \rangle }_{\varphi }=\frac{1}{2\pi }\int_{0}^{2\pi }\left(\cdots \right)\text{d}\varphi$. The statistics of Ω^2^ as a function of wave phase were investigated considering regular waves of mean steepness *ϵ* = 0.08, case *C3*. The phase *φ* is defined crest-to-crest on [0, 2*π*〉. To find *φ*(*t*), the time series of spanwise-averaged surface elevation from the LIF data, *η*(*t*) ≡ 〈*η*’〉_*y*_(*t*) was calculated for each ensemble, as *φ*(*t*) = arg[*H*(*t*)], where *H*(*t*) is the (complex) analytic signal of *η*(*t*) whence $\bar{{\text{Ω}}^{2}}(z,\varphi )\equiv {\langle \bar{{\text{Ω}}^{'2}}\rangle }_{y}$ and $\bar{\eta }(\varphi )$ were obtained by ensemble averaging in bins of *z* and *φ*.

**Figure 3:**
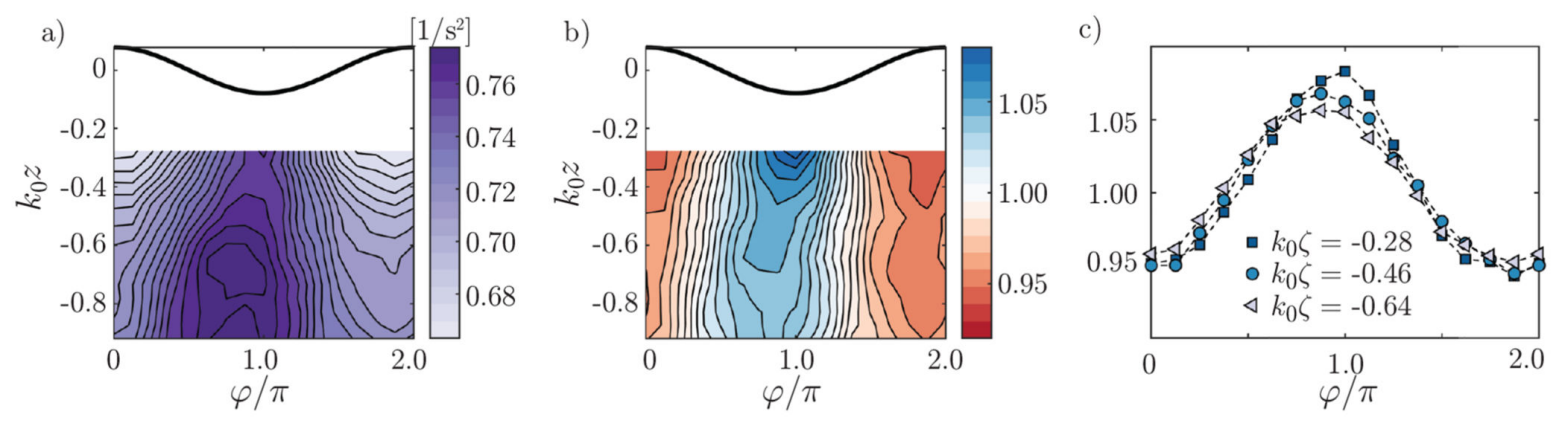
Ensemble–averaged cross-plane enstrophy in regular waves (case C3). (*a*) $\bar{{\text{Ω}}^{2}}\left(z,\varphi \right)$; (*b*) $\bar{{\text{Ω}}^{2}}\left(z,\varphi \right)/{\langle \bar{{\text{Ω}}^{2}}\rangle }_{\varphi }\left(z\right)$ (Eulerian reference frame); (*c*) $\bar{{\text{Ω}}^{2}}\left(\zeta ,\varphi \right)/{\langle \bar{{\text{Ω}}^{2}}\rangle }_{\varphi }\left(\zeta \right)$ (surface–following reference frame), for values of $k_{0}\zeta =k_{0}\left(z-\bar{\eta }\left(\varphi \right)\right)$ given in the legend.

A plot of $\bar{{\text{Ω}}^{2}}(z,\varphi )$ is shown in figure 3a) with $\bar{\eta }(\varphi )$ shown as a solid black line. There is a cleartrend that the absolute cross–plane enstrophy at a constant depth is enhanced(decreased) under troughs (crests) due to streamwise stretching and compression of vortices by the wave orbital motion. Due to the *z*-dependence of $\bar{{\text{Ω}}^{2}}$, the relative quantity $\bar{{\text{Ω}}^{2}}(z,\varphi )/{\langle \bar{{\text{Ω}}^{2}}\rangle }_{\varphi }(z)$ gives a clearer interpretation of the wave–driven vorticity oscillation, shown in 3b).

Figure 3a is in excellent agreement with figure 7a of Guo & Shen (2013) from direct numerical simulations with similar wave steepness, including the positions of maxima and minima, the shape of contours and the relative variation in magnitude. A curious observation in figure 3 is that enstrophy variations undergo a depth-dependent phase shift, which the simulations do not appear to show.

A turbulent vortex being strained by the wave motion will be convected in orbits so that its distance from the surface is near constant. It is instructive therefore to also consider a surface-following coordinatee system, $z\to \zeta =z-\bar{\eta }(\varphi )$. A plot of normalised cross-plane enstrophy $\bar{{\text{Ω}}^{2}}(\zeta ,\varphi )/{\langle \bar{{\text{Ω}}^{2}}\rangle }_{\varphi }(\zeta )$ is shown in figure 3c) for three values of *k*_0_*ζ*. The same trends are observed, providing further evidence that these phenomena cannot simply be ascribed to the variation of $\bar{{\text{Ω}}^{2}}$ with depth, shifted by the wave motion, since such an effect would not be visible in the surface-following system.

**Figure 4:**
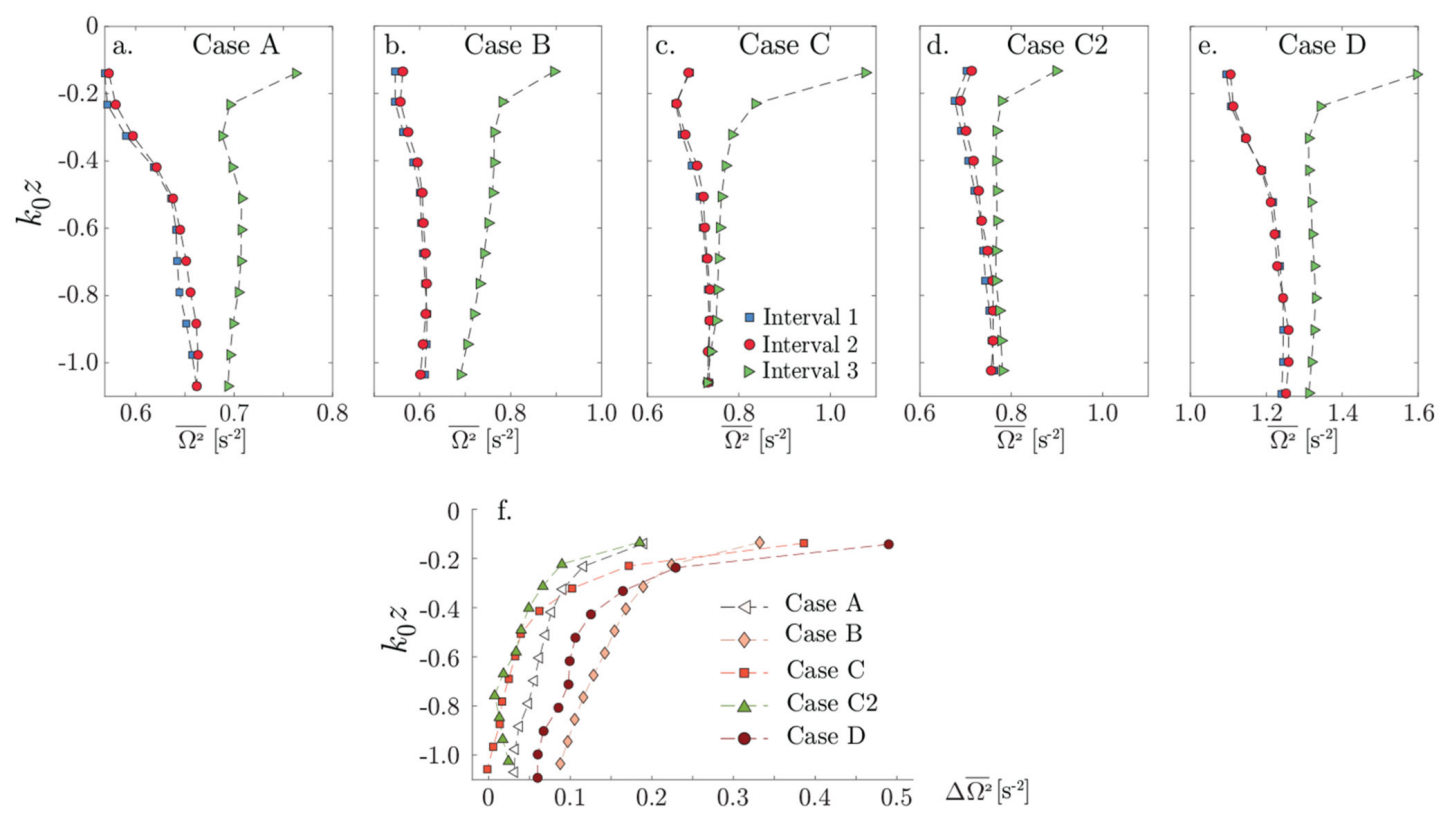
(a-e) Cross–plane enstrophy $\bar{{\text{Ω}}^{2}}(z)$ for cases and time intervals as indicated. (f) Measured increase of $\bar{{\text{Ω}}^{2}}(z)$ from interval 2 to 3 for all cases.

We next turn to the cumulative influence of the wave groups on the streamwise vorticity over many wave periods, of order *ϵ*^2^*t* according to theory. We compare $\bar{{\text{Ω}}^{2}}(z)\equiv {\langle \bar{{\text{Ω}}^{'2}}\rangle }_{yt}$ measured in each of the three measurement intervals relative to the wave group (figure 2b). Measured values of $\bar{{\text{Ω}}^{2}}(z)$ are shown in figure 4 for four different turbulence characteristics, cases *A*-*D*. In all cases $\bar{{\text{Ω}}^{2}}(z)$ is essentially identical in *I*_1_ and *I*_2_, but clearly increased in interval *I*_3_, most strongly near the surface. Intervals *I*_2_ and *I*_3_ correspond to the leading and trailing edges of the wave group, respectively, and thus contain some wave orbital motion; the negligible difference between *I*_1_ and *I*_2_ provides confidence that the increase of $\bar{{\text{Ω}}^{2}}(z)$ in *I*_3_ is due to the cumulative effect of wave-turbulence interactions rather than a spurious mapping of wave motion to vorticity. The results are qualitatively consistent with theoretical predictions. In particular, the rapid decrease of the final enstrophy with depth, more rapid than the ∼ *e*^2*k*_0_*z*^ dependence of the Stokes drift, is also predicted by Teixeira & Belcher (2002) due to the blocking effect of the free surface.

The increase in $\bar{{\text{Ω}}^{2}}(z)$ from *I*_2_ to *I*_3_ is shown in figure 4(f) for all flow cases. While the magnitude of the increase varies from case to case, the depth dependence is highly similar, with a gentle, roughly linear increase up to *k*_0_*z* ≈ 0.3, from which point it increases very rapidly towards the surface. This closely resembles general depth dependence which Guo & Shen (2013) observe (their figure 12) when considering the term which corresponds to streamwise tilting of vertical vorticity in the Lagrangian-averaged vorticity evolution equation. Comparing cases *C* and *C*2 shows that higher steepness leads to a larger increase near the surface, as expected (differences between the two cases in the deeper region are too small for conclusions to be drawn given the uncertainty level). On the other hand, the relative increase in $\bar{{\text{Ω}}^{2}}(z)$ from *I*_2_ to *I*_3_ shows no obvious trend based on these 5 cases.

The observations in Figure 4 pose several further questions. The increase in streamwise turbulent enstrophy will not remain linear in time indefinitely under continued wave forcing, but will eventually reach an equilibrium state. The lack of a simple relationship between initial and final enstrophy might indicate that saturation has to some extent occurred, yet the properties of a hypothetical asymptotic state, how it depends on wave and initial turbulence conditions, and to what extent it has been reached in our experiments, are not known.

### 3.2 Angular wave scattering by turbulence

To analyse the wave angle of propagation we consider the surface elevation time series from each of the eight wave probes labelled *p* = 1…8 (see figure 1). For each pair of parallel probes *p* and *p* + 1 (odd *p*) a phase difference was computed, Δ*φ_y_*(*t*) = arg [*H_p_*(*t*)*H*_*p*+1_ (*t*)*], where *H_p_* is the analytic signal of probe *p* and (*) denotes the complex conjugate. The spanwise phase difference Δ*φ_y_*(*t*) was found to vary slowly in time during the passage of a wave group, and was interpreted as being due to the wave propagating at mean angle *θ* ≈ Δ*φ_y_*/(*k*_0_Δ*y*) to the *x* axis, where Δ*y* = 1.2 m is the spanwise interprobe distance.

**Figure 5:**
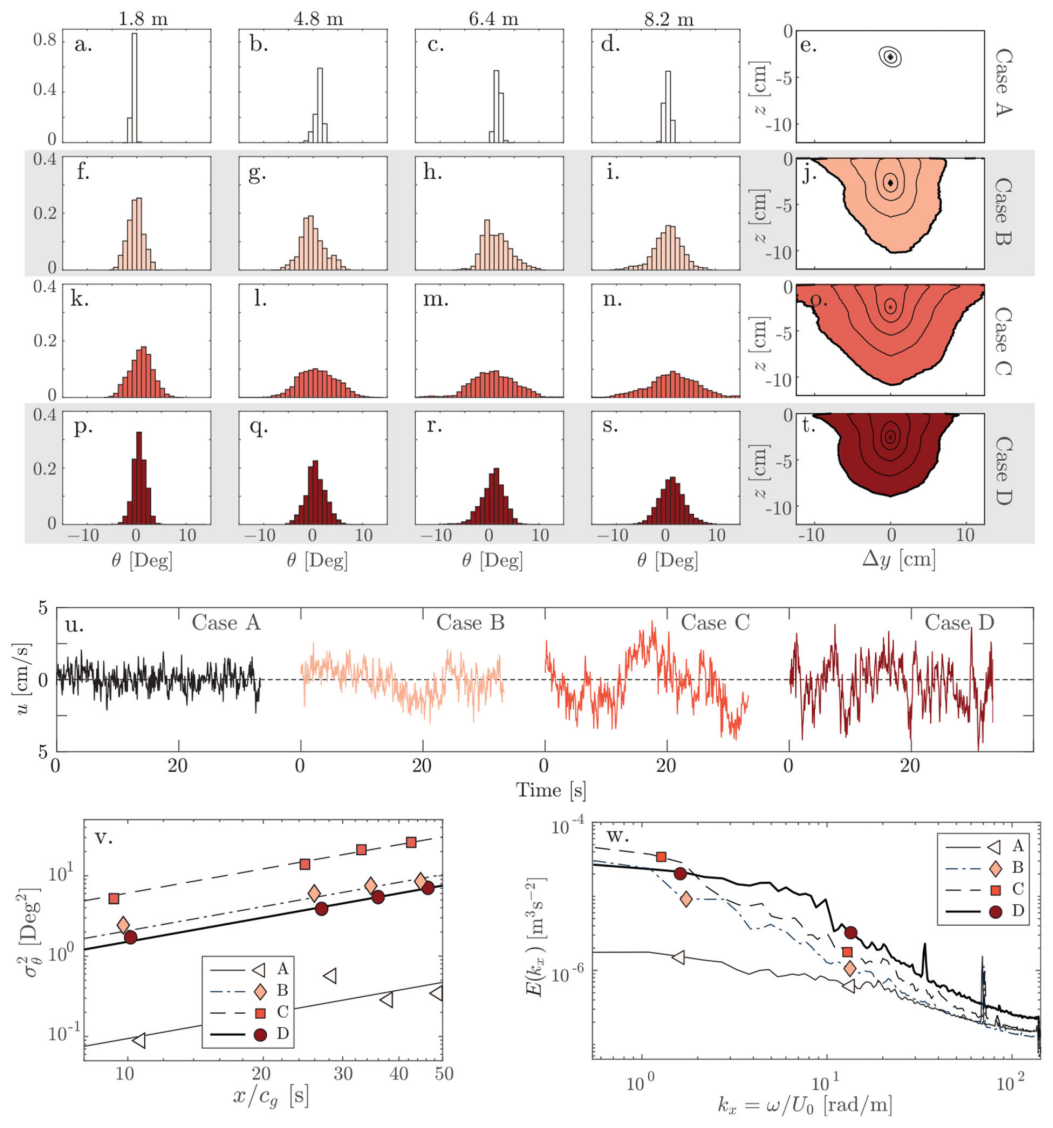
(a-d,f-i,k-n,p-s) Histograms of the wave angular PDF; the distance from wavemaker to wave probe is shown above each column, rows correspond to cases *A* through *D*. (e,j,o,t) Contours of the streamwise velocity autocorrelation *R_uu_* = 0.9, 0.8, 0.7, 0.6, 0.5 in the spanwise plane around a point at depth 26 mm; u) Example time series of *u*‘(0, *z*_ref_, *t*); v) Variance of the wave directional spreading as a function of the travel time in log-log scale; The dashed lines are linear fits with slope $D_{\theta \theta }^{\text{exp}}$ assuming $\sigma_{\theta }^{2}=0$ at *x* = 0; the markers show measured values. w) Power spectral density of the streamwise velocity; markers are here merely an aid to distinguish the graphs.

Figure 5 (a-d, f-i, k-n p-s) shows the ensemble-averaged probability density function (PDF) of the wave angle *θ* for cases *A*-*D* and corresponding variances as a function of group propagation time *x*/*c_g_* are shown in figure 5v. The histograms and symbols are colour coded from dark to light in order of increasing turbulence intensity (see table 2). Strikingly, case *D* with the highest turbulence intensity does not have the greatest rate of directional spreading. Instead, in our four cases the rate of spreading increases monotonously with increasing integral scale. Eddy size itself cannot in general determine the scattering rate; a physically more reasonable hypothesis is that scattering increases with the turbulent energy content at the longest length scales. A plot in figure 5w of the turbulent power spectrum, averaged over the field of view and converted to streamwise conjugate length using Taylor’s frozen eddy hypothesis, is consistent with such a conclusion. (We note that we are basing our analysis on measurements of the turbulence at 83.8*M* downstream of the active grid; Jooss *et al*. (2021) found the turbulence intensity and length scale to vary somewhat with distance downstream from the grid.)

Further insight is gained by investigating the spatial correlation for the streamwise velocity component *u*’ which affects the waves the most. In the manner of Christensen & Adrian (2001) we plot contours from 0.9 to 0.5 of the streamwise two-point autocorrelation *R_uu_*(Δ*y*, *z*) = 〈*u*’(*y*, *z*_ref_)*u*’(*y* + Δ*y*, *z*)〉_*yt*_/*u*_∞_(*z*_ref_)*u*_∞_(*z*) in figure 5e,j,o,t, where *z*_ref_ = -26mm is a reference depth. Due to relatively short time series, statistics are limited, but the qualitative picture is very telling: spanwise correlation lengths are considerably longer for case *C* than for *B* and *D*, corresponding to broader energy-carrying turbulent eddies. An estimate of the streamwise integral scale $L_{x}^{x}$ can be obtained from the variance and mean of the time Δ*τ* between consecutive zeros of *u*’(*y*, *z*_ref_, *t*) in the 15 Hz data; an example time series of *u*’ for each case is shown in figure 5u. Using the approach of Mora & Obligado (2020), we use as integral scale $L_{x}^{x}=\frac{1}{4}\pi U_{0}\text{Var}(\text{Δ}\tau )/{\langle \text{Δ}\tau \rangle }_{t}$. The procedure was performed for each point (*y*, *z*_ref_) in the field of view and averaged, with values for cases *A*-*D* as listed in table 2 (note, the same trends are found for any choice of point(s) (*y*, *z*) in the field of view). Again case *C* displays the longest structures. Power spectra of the time series (figure 5w) illustrates the same: case *D* has the greatest TKE in total, but at the very longest scales, *C* is more energetic. The clear indication is thus that wave scattering is determined to a greater extent by the energy of the turbulence at the larger scales measured here rather than total integrated energy.

At a qualitative level this is consistent with theoretical predictions of Phillips (1959) and Fabrikant & Raevsky (1994). Suzuki (2019) finds that long but thin streamwise rolls and streaks can refract waves, indicating that also eddy-size dependence in spanwise and vertical directions should be investigated in the future.

We are not aware of any theory which allows quantitative comparison with our results (e.g. Fabrikant & Raevsky (1994) requires measurement of the vertical vorticity spectrum). As a qualitative test we consider the formula derived by Smit & Janssen (2019): $D_{\theta \theta }^{\text{theory}}=\frac{1}{c_{0}}\int_{0}^{\infty }kE(k)\text{d}k$, where *D_θθ_* is a directional diffusion coefficient, $c_{0}=\sqrt{gk_{0}}$, and *E*(*k*) is the velocity power spectral density (PSD). This expression, however, is based on the WKB approximation and assumes that turbulent eddies are large compared to a wavelength, an assumption which is not in general satisfied in our experiment, so we cannot hope for quantitative agreement. Note, however, that case *C* has the highest PSD when the “turbulence wavenumber” *k_x_* = *ω*/*U*_0_ (*ω* is reciprocal time) is ∼ 1 rad/m, considerably smaller than *k*_0_ ∼ 9 rad/m, hence the WKB approximation may not be entirely unreasonable in a scattering context. When inserting lab data into the $D_{\theta \theta }^{\text{theory}}$ expression, we note that the spanwise distance between the wave probes imposes a Nyquist wavenumber *π*/Δ*y* ≈ 2.62 rad/m on the wave angles, which we take as the upper integral limit.

The measured directional variance values $\sigma_{\theta }^{2}=\text{Var}(\theta )$ seem to increase linearly as a function of propagation time *x*/*c_g_*, at least in cases *B*-*D*, indicating that the scattering can be modelled as a diffusion process despite being outside the expected range of applicability of WKB theory and turbulence characteristics not being entirely constant with distance from the grid. Measured diffusion coefficients $D_{\theta \theta }^{\text{exp}}$ were found by fitting $\sigma_{\theta }^{2}$ to a linear function $xD_{\theta \theta }^{\text{exp}}/c_{g}$, shown as lines in figure 5v and listed in table 2.

The theoretical values for cases *A*, *B*, *C*, *D*, calculated using the measured spectra in figure 5w, are $D_{\theta \theta }^{\text{theory}}=(0.014,\;0.14,\;0.26,\;0.20){\text{Deg}}^{2}/\text{s}$, in reasonable agreement with, and adhering to the trend of, the fit of the values to the data, ${\text{D}}_{\theta \theta }^{\text{exp}}$. These values carry considerable uncertainty, being sensitive to the spectra at the very lowest frequencies that our experiment can resolve. That a theory assuming velocity variations be larger than a wavelength is in even rough agreement seems to indicate once more that smaller turbulence length scales are of lesser importance; given the uncertainty and suspect assumptions, however, this is perhaps best considered a curiosity at present, and further theoretical and experimental investigations are required to confirm the behaviour.

## 4 Conclusions

In the presence of waves, the statistics of the streamwise turbulent vorticity component showed a clear dependence on the wave phase, with vorticity being increased under the troughs and decreased under the crests at a fixed depth, both in a static (lab) and a surface-following reference frame. The results confirm previous theory and numerical simulations where the variation was attributed to the periodic stretching and straining of vortical structures due to the orbital motion.

By comparing the streamwise enstrophy (mean-square of streamwise vorticity) before vs after the passage of wave groups, a strong enhancement is observed in all cases. The difference is found to grow with increasing wave steepness as expected, but the gain in enstrophy caused by the passage of the wave group shows no simple dependence on the properties of the initial turbulence.

The converse effect, the scattering of waves by a turbulent velocity field, is also studied. The variance of the wave angle of initially unidirectional waves was found to increase as a function of propagation distance upstream, corresponding to directional spreading of the wave spectrum. The rate of angular broadening was found to be greatest for the turbulent case containing the largest energy-carrying turbulent structures, not the case with the greatest total TKE overall.

In our experiments, turbulent scales large compared with a wavelength scatter waves most efficiently, whereas wave energy is transferred to turbulent scales well below a wavelength. We thus highlight the importance of understanding the turbulent cascade under surface wave forcing. Bearing limitations in mind, our observations lend support to the notion that wave intensification of turbulence can be described in terms of vorticity, whereas scattering is primarily a process of diffraction and refraction due to fluctuations of horizontal velocity, not vorticity as such.

## Figures and Tables

**Table 1:** Test case parameters

Case	$\bar{f_{G}}[\text{Hz}]$	*S*_0_ [Deg]	*τ*_wm_[s]	*T*_wm_[s]	*T*_PIV_[s]	*N*_int_	*N*_ens_
A	0	18	6	24	10	3	60
B	1.5	18	6	24	10	3	60
C	1.5	18	6	24	10	3	60
D	0.2	18	6	24	10	3	60
C2	1.5	12	6	24	10	3	20
C3	1.5	5	20	32	40	1	24

**Table 2:** Measured turbulence and wave parameters

Case	*U*_0_ [m/s]	*k*_0_ [rad/m]	*λ*_0_ [m]	[*u*_∞_, *V*_∞_, *W*_∞_]/*U*_0_ ×10^-12^	*Fr*^2^ ×10^-4^	$L_{x}^{x}$ [m]	*a*_0_ *k*_0_	*τ*_0_ [s]	$D_{\theta \theta }^{\text{exp}}$ [Deg^2^/s]
A	0.34	9.5	0.66	[2.1, 2.0, 1.7]	0.5	0.051	0.20	2.4	0.009(5)
B	0.33	9.2	0.68	[3.3, 3.0, 2.2]	1.1	0.26	0.20	2.6	0.21(4)
C	0.33	9.0	0.70	[4.7, 3.6, 2.8]	2.3	0.32	0.22	2.9	0.61(4)
D	0.34	9.3	0.68	[5.1, 4.9, 3.7]	2.8	0.20	0.22	2.8	0.15(1)
C2	0.33	8.9	0.71	[5.1, 3.6, 2.8]	2.5	∼ *C*	0.15	2.9	-
C3	∼ C2	∼ C2	∼ C2	∼ C2	∼ C2	∼ C	0.08^†^	-	-

The Froude number is *Fr*^2^ ≡ (*u*_∞_)^2^*k*_0_/*g*.

† A dagger (†) denotes average value.

For the diffusion coefficients $D_{\theta \theta }^{\text{exp}}$ the values in parentheses indicate the uncertainty of the last digit.
